# Cancer-Associated Fibroblasts in Oral Cancer: A Current Perspective on Function and Potential for Therapeutic Targeting

**DOI:** 10.3389/froh.2021.686337

**Published:** 2021-07-01

**Authors:** Kamila J. Bienkowska, Christopher J. Hanley, Gareth J. Thomas

**Affiliations:** ^1^School of Cancer Sciences, University of Southampton, Southampton, United Kingdom; ^2^Cancer Research UK and National Institute for Health Research (NIHR) Southampton Experimental Cancer Medicine Centre, Southampton, United Kingdom

**Keywords:** oral cancer, head & neck squamous cell carcinoma, tumour microenvironment, cancer-associated fibroblasts, myofibroblasts

## Abstract

The role of the tumour microenvironement (TME) in cancer progression and resistance to therapies is now widely recognized. The most prominent non-immune cell type in the microenvironment of oral cancer (OSCC) is cancer-associated fibroblasts (CAF). Although CAF are a poorly characterised and heterogenous cell population, those with an “activated” myofibroblastic phenotype have been shown to support OSCC progression, promoting growth, invasion and numerous other “hallmarks of malignancy.” CAF also confer broad resistance to different types of therapy, including chemo/radiotherapy and EGFR inhibitors; consistent with this, CAF-rich OSCC are associated with poor prognosis. In recent years, much CAF research has focused on their immunological role in the tumour microenvironment, showing that CAF shield tumours from immune attack through multiple mechanisms, and particularly on their role in promoting resistance to anti-PD-1/PD-L1 checkpoint inhibitors, an exciting development for the treatment of recurrent/metastatic oral cancer, but which fails in most patients. This review summarises our current understanding of CAF subtypes and function in OSCC and discusses the potential for targeting these cells therapeutically.

## Introduction

Historically, the search for novel therapies has focused on tumour cells, attempting to inhibit oncogenic pathways that drive tumour progression; targeting the EGFR pathway for example. Although such targeted therapies can produce dramatic initial results, acquired resistance, where tumours progress after initial response, seems an almost inevitable consequence of this approach. In recent years, and particularly following the success of anti-PD-1/PD-L1 (programmed cell death protein 1/programmed death ligand 1) checkpoint immunotherapy, there has been a realisation that a tumour is a complex mixture of different cell types that interact to promote tumour progression, and this has generated significant interest in developing therapies that target the tumour microenvironment (TME) [1].

## CAF Heterogeneity

The most prominent non-immune cells within cancers are cancer-associated fibroblasts (CAF); these can account for up to 80% of tumour mass in late stage head & neck cancers (HNSCC) and are generally assumed to be tumour-promoting [2, 3]. CAF remain a poorly characterised and heterogenous cell population; most studies have focused on myofibroblastic CAF and the terminology within the literature has been confusing with “cancer-associated fibroblast,” “myofibroblast” and “peritumoral fibroblast” variably used, and only recently is the term myofibroblastic CAF (myCAF) starting to be used consistently in the literature. These contractile, α-SMA-positive cells are generated principally through TGF-β signalling/mechanotransduction [4], and secrete extracellular matrix (ECM) analogous to myofibroblasts found in healing wounds and fibrotic disorders. In tissue sections these are usually identified using immunochemistry for α-SMA, although this protein is also expressed by pericytes [5] and smooth muscle cells [6]. Indeed, there is no specific single marker that accurately identifies CAF; other markers such as FAP-α (fibroblast activation protein α), FSP1 (fibroblast specific protein 1) and PDGFRB (platelet derived growth factor receptor β) have been used to identify CAF, but also are not CAF specific [7]. Moreover, and despite extensive research, the myCAF cell of origin also remains inconclusive, and, although it is thought that most myCAF originate from local fibroblasts–pericytes, adipocytes, endothelial cells and bone marrow-derived mesenchymal stem cells have all been shown to be potential myCAF progenitors [8, 9]. In recent years, macrophages and cancer stem cells have also been highlighted as potential myCAF precursors [1, 10, 11], and it remains unclear whether cell of origin affects the final myCAF phenotype.

The advent of single cell transcriptomic sequencing (scRNA-seq) is beginning to characterise CAF heterogeneity within HNSCC and other cancers, and it is now accepted that not all CAF subpopulations are characterised by high expression of α-SMA, and conversely, not all α-SMA-positve CAF are myofibroblastic. Elyada et al. [12] and Öhlund et al. [13] analysed pancreatic cancers using scRNA-seq and identified two main CAF populations; myCAF and a subpopulation characterised by expression of inflammatory genes, which were termed iCAF. iCAF subpopulations have subsequently also been identified in breast cancer [14]. Puram et al. [15] used scRNA-seq to characterise HNSCC and described two main fibroblast groups which were termed “myofibroblasts” and “CAF.” The latter group could be further divided into two subclusters through differential expression of immediate early response genes, mesenchymal markers, ligands and receptors, and ECM proteins, suggesting further potential subdivisions. It is not yet clear whether iCAF are found in HNSCC as a distinct subpopulation. Patel et al. [16] performed transcriptomic analysis on CAF cultures from oral cancers (OSCC) and also identified two main subgroups: CAF1 (α-SMA^low^) and CAF2 (α-SMA^high^). CAF1 was associated with increased proliferation of cancer cells but with suppressed self-renewal growth of oral stem-like cancer cells (oral-SLCCs). Conversely, CAF2 correlated positively with frequency of oral-SLCCs but negatively with tumour cell proliferation. BMP4 (bone morphogenetic protein 4) was differentially expressed between the two CAF populations and was suggested to be at least partially responsible for exerting the suppressive effect on cancer cells' stemness. Costea et al. [17] compared heterogeneity of OSCC CAF with normal fibroblasts, performing transcriptomic analysis on CAF cultured in 2D and 3D-matrices. This study also identified two CAF populations; CAF-N, motile fibroblasts whose transcriptome and secretome were more similar to normal fibroblasts, and CAF-D subpopulation, which had a more divergent expression pattern and secreted high levels of TGF-β1. Both CAF subtypes resulted in higher tumour incidence and deeper invasion in murine models, though intriguingly, CAF-N was best at supporting tumour formation. The two subpopulations were not compared in terms of their α-SMA expression, however 50% of the upregulated genes compared with normal fibroblasts were TGF-β targets, suggesting differentiation towards a myofibroblast-like phenotype. The authors suggest that the two populations may be a spectrum in CAF development, with CAF-N representing an earlier stage of differentiation.

Fibroblast phenotype may also vary according to molecular phenotypes of HNSCC. Hassona et al. [18] compared CAF from genetically stable OSCC (GS-OSCC; maintaining wild-type p53) and genetically unstable OSCC (GU-OSCC) and found that cultured CAF from GU-OSCC were significantly more senescent. They found that malignant keratinocytes from GU-OSCC produce high levels of reactive oxygen species (ROS), associated with increased production of TGFβ1 and TGFβ2, and (myo)fibroblast activation. Consistent with this observation, senescent fibroblasts commonly express α-SMA, are contractile and tumour promoting [19, 20] but differ from myofibroblasts in their ability to deposit ECM [19, 21]. Notably, the generation of intracellular reactive oxygen species plays a major role in CAF (and myofibroblast) differentiation, with the ROS-producing enzyme, NADPH oxidase 4 (NOX4), central to this process [22, 23].

However, despite the progress made in identifying different CAF subtypes in oral cancer (Table 1) characterisation of CAF heterogeneity remains incomplete; studies have identified CAF-secreted inflammatory factors as promoting OSCC progression, but it is not yet clear whether iCAF are a distinct subpopulation in HNSCC or whether myCAF also acquire inflammatory properties in certain situations. As novel CAF subgroups are identified, their effect on OSCC progression will be an intriguing area of research, and in order to study function it will be necessary to modify *in vitro* culture conditions to maintain these phenotypes. Standard 2D-tissue culture techniques tend to skew CAF to a myCAF-like phenotype [24], meaning that studies have tended to focus, either deliberately or inadvertently, on myCAF. Future experimental work will require a more detailed characterisation of CAF phenotypes given increasing understanding of CAF heterogeneity and plasticity.

**Table 1:** Heterogeneity of CAF in oral cancer.

**Source**	**Tumour**	**CAF subpopulations**	**Description/Markers**
Puram et al. [15]	HNSCC (oral cavity)	Myofibroblasts	ACTA2, MCAM, MYLK, MYL9, IL6, PDGFA
		CAF	FAP, THY1, PDPN, MMP2, MMP11, PDGFRA/L, TGFB3. Could be further divided into CAF1 and CAF2
		Resting fibroblasts	Lack of activation markers
Patel et al. [16]	Gingivobuccal–oral tumour	CAF1 (α-SMA^low^BMP4^+ve^)	Increased proliferation of cancer cells; supressed self-renewal growth of oral-SLCC
		CAF2 (α-SMA^high^BMP4^−*ve*^)	Negative correlation with cancer cells' proliferation; increased frequency of oral-SLCC
Costea et al. [17]	OSCC	CAF-N	Motile; high production of hyaluronan; promoted invasion; secretome similar to the secretome of normal fibroblasts;
		CAF-D	Less motile; high expression of TGF-β1; promoted invasion and EMT; secretome different from the secretome of normal fibroblasts
Hassona et al. [18]	GS-OSCC	Non-senescent CAF	Non-senescent CAF failed to promote keratinocytes' invasion *in vitro*
	GU-OSCC	Senescent CAF	Malignant keratinocytes induced fibroblast activation and senescence through ROS and TGF-β; senescent CAF promoted keratinocytes' invasion *in vitro*

## CAF and OSCC Survival

Numerous studies have shown that CAF-rich OSCC are associated with significantly shorter patient survival. In the largest study of this type, Marsh et al. [25] performed a retrospective analysis of 282 OSCC patients, analysing a number of tumour cell and stromal cell molecular markers by IHC. They found that the best independent risk factor of early OSCC death was high stromal α-SMA expression, which produced the highest adjusted hazard ratio (HR 3.06, 95% CI 1.65-5.66; *p* = 0.002), and likelihood ratio (3.6; Detection rate:False positive rate) of any feature examined, and was strongly associated with mortality regardless of disease stage. A recent meta-analysis examined correlations between myCAF (assessed by α-SMA immunochemistry) and risk of OSCC death, analysing 1,328 patients from 12 studies [26]. The combined results showed that myCAF predicted poor overall survival (HR 2.16 95% CI 1.60–2.92; *P* < 0.00001) and shortened disease-free survival (HR 3.32 95% CI 2.09–5.26; *P* < 0.00001). The presence of high levels of stromal myCAF was associated with pathological features related to tumour aggressiveness, including depth of invasion, lymphatic invasion and extra-nodal metastatic spread [26].

## CAF and Malignant Transformation

Oral potentially malignant disorders (PMDs) include leukoplakia, erythroplakia and oral submucous fibrosis (OSMF) [27]. Approximately 1% of potentially malignant disorders give rise to cancer but the mechanism of the transformation is poorly understood [28]. Changes in stroma, such as myofibroblast activation, are considered as potential promoters of the transformation of pre-malignant lesions. Several studies, using immunohistochemistry staining for α-SMA, have compared the number of myofibroblasts in potentially malignant disorders and OSCC. Generally, studies report a lack of myofibroblasts in leukoplakias and erythroplakias, including a meta-analysis that analysed 19 articles [28, 29]. However, some studies have reported increased myofibroblasts in high-risk epithelial dysplasia [30]. Conversely, studies have consistently reported increased myofibroblasts in OSMF compared to healthy oral mucosa [28, 31–33], with late-stage OSMF reported to contain more myofibroblasts than early-stage OSMF [32]. These findings are perhaps not surprising given that OSMF is a fibrotic disorder, and whether myofibroblasts play an active role in the malignant transformation of this disease remains to be determined.

## CAF Function in OSCC

**Figure 1:**
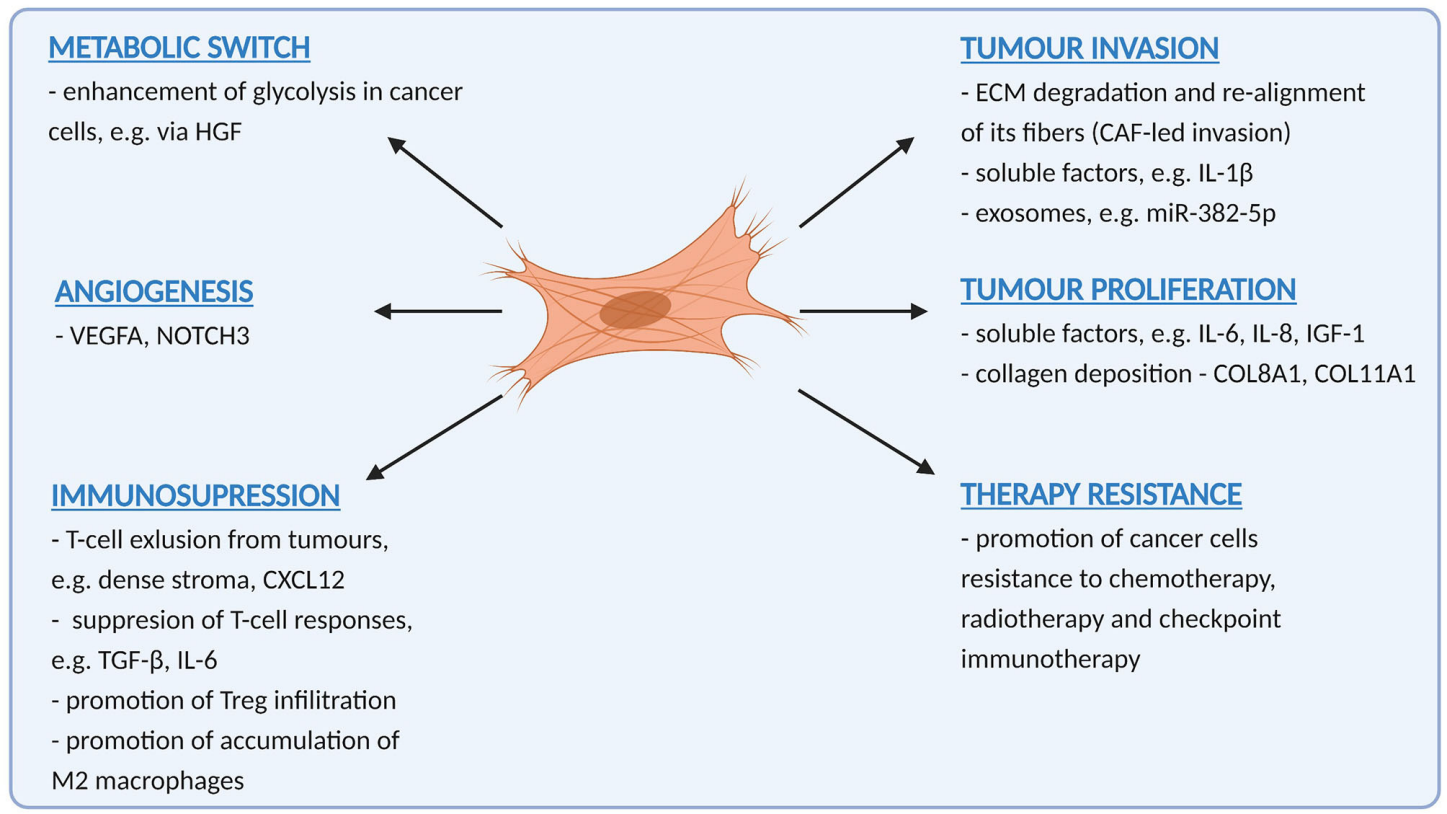
A summary of pro-tumoural functions exerted by cancer-associated fibroblasts in oral cancer (Created with BioRender.com).

Clues as to how CAF function to promote OSCC progression can be found in clinic-pathological correlates. The fact that CAF correlate with many features of tumour aggressiveness, including invasion, metastasis, absence of T-cells and therapy resistance suggest that their role is multifactorial, and this is borne out in numerous functional studies that show that CAF promote many, if not all of the “hallmarks of malignancy” [34]. Understanding these functions will help develop CAF-targeting strategies for OSCC treatment. Tumour promoting functions of CAF in OSCC are summarised in Figure 1.

## CAF and Tumour Cell Invasion

CAF promote tumour invasion through multiple mechanisms, including deposition and remodelling of ECM, a central myCAF function. Tumour ECM forms a dense meshwork, generally difficult for cells to penetrate. Gaggioli et al. [35] found that CAF create “tracks” in ECM through which tumour cells invade and are led by CAF. Generation of tracks was dependent on a combination of protease- and force-mediated matrix remodelling mechanisms. Expression of integrins α3 and α5, and activity of Rho and ROCK (Rho-associated protein kinase) (drivers of a force-mediated remodelling) in fibroblasts were shown to be crucial for the remodelling process. More recently, Chen et al. [36] demonstrated that HNSCC cell invasion is facilitated by fibroblast-dependent degradation of dense ECM. By varying collagen concentration in co-culture experiments, they found in lower concentrations of collagen (2 mg/ml), that both tumour cells and fibroblasts remodel the matrix and invade into the surrounding collagen independently. However, tumour cells growing in more concentrated collagen (8 mg/ml) were unable to penetrate the matrix unless fibroblasts were present. Similar to the Gagglioli study, fibroblasts were seen to lead cancer cells through the matrix. Both studies suggest that cancer cells require fibroblasts to remodel dense extracellular matrix in order to invade into surrounding tissues and disseminate.

A similar mechanism has been reported to regulate OSCC invasion into bone [37], where a fibrous CAF stroma characterised by a high expression of α-SMA was observed to sit between tumour cells and bone. Notably, α-SMA-positive CAF were commonly seen infiltrating bone ahead of cancer cells, again suggesting that they are the leading cells in cancer invasion. Two proteins associated with regulation of bone resorption: RANKL (receptor activator of NF κ-B ligand) and OPG (tumour necrosis factor receptor superfamily member 11B) were expressed in OSCC cells and bone-adjacent stroma. Moreover, treatment with conditioned media from both experimentally generated CAF and OSCC-tumour-derived CAF could induce osteoclastogenesis in macrophages – i.e., a transdifferentation of macrophages into bone-resorbing osteoclasts. CAF induced osteoclastogenesis in macrophages to a greater extent than cancer cells, suggesting they may be the main orchestrators of bone invasion in OSCC, modulating macrophages as well as tumour cells in this process.

CAF produce numerous ECM proteins and the composition of the matrix can significantly affect cancer invasion. For example, CAF in OSCC express high levels of hyaluronan synthetase 2 (HAS2), an enzyme producing hyaluronan, which is a major component of ECM. HAS2 promotes invasion of OSCC cells, and its expression correlates with advanced clinical stages and cervical lymph node metastases [38]. The mechanism underlying HAS2-dependent invasion of cancer cells was suggested to depend on regulation of the balance between ECM-degrading matrix metalloproteinases (MMPs) and their inhibitors (tissue inhibitors of metalloproteinases; TIMPs). HAS2^+^ CAF expressed higher levels of MMP1 and lower levels of TIMP1 than normal fibroblasts, and knockdown or inhibition of HAS2 in CAF not only reduced OSCC invasion but also reversed the ratio between MMP1 and TIMP1 expression, reducing expression of MMP1 and upregulating TIMP1. HAS2 has also been reported to play a role in regulating the fibroblast response to TGF-β. Dermal fibroblasts and fibroblast from the oral mucosa respond differently to TGF-β1 treatment [39, 40], which promotes proliferation in dermal fibroblasts but suppresses proliferation in fibroblasts from the oral mucosa. Overexpression of HAS2 in oral fibroblasts resulted in a pro-proliferative response to TGFβ1 stimulation. The presence of hyaluronan in the TME is also known to promote cancer cell and CAF motility [41]. A subtype of hyaluronan-producing CAF could represent a promising drug target.

CAF also modulate tumour cell invasion via secreted soluble factors. Various CAF-secreted factors, have been implicated in promoting HNSCC invasion and/or proliferation, including IL-1β (interleukin 1β) [42], activin A [43], HGF (hepatocyte growth factor) [44] and EREG (epiregulin) [45], which has additionally been suggested to play an autocrine role in CAF activation [45]. In recent years there has also been significant interest in CAF-tumour cell communication via exosomes. These secreted extracellular vesicles contain proteins, lipids and nucleic acids, such as messenger RNA (mRNA), micro RNA (miRNA), long non-coding RNA (lncRNA) and others [46], and exosomal miR-382-5p and MFAP5 have been shown in separate studies to promote OSCC cell migration and invasion [47, 48]. Intriguingly, Li et al. [49] showed miR-34a-5p could suppress proliferation and metastasis of OSCC cells and that CAF exosomes contained reduced levels of this miRNA, suggesting that the cancer-promoting features of exosomes might not only be due to a transfer of pro-tumoral factors but also due to the lack of suppressive factors.

## CAF and Tumour Proliferation

CAF have been shown to promote proliferation of cancer cells in multiple tumour types [50–52]. Co-injection of CAF with HNSCC cells *in vivo* enhances tumour growth [53], and similar observations have been made in 3D co-culture models, where CAF increase proliferation of tumour spheroids [54]. Conditioned medium from CAF promotes HNSCC cell proliferation *in vitro* [53] and consistent with this a number of secreted cytokines and growth factors have been shown to stimulate tumour growth. For example, interleukin-6 (IL-6) has been shown to upregulate expression of osteopontin (SPP1) in HNSCC cells and to increase their proliferation via integrin/NF-κB signalling [55]. Osteopontin may play an important role in bi-directional communication between CAF and HNSCC cells, and in breast cancer models has been shown to induce transformation of mesenchymal stem cells into CAF, in a MZF1(myeloid zinc finger 1)-TGFβ1-dependent manner [56]. Plasma osteopontin has also been suggested as a potential prognostic biomarker in HNSCC patients; negatively correlating with overall and relapse-free survival [57].

Another example of a reciprocal paracrine interaction between tumour cells and CAF was shown by Bae et al. [58], who observed that tumour volume of orthotopic tumours correlated with numbers of CAF co-injected with tumour cells. They found that interleukin-1α (IL-1α) secreted by OSCC cells increased proliferation of CAF and upregulated expression of secretory cytokines, including CCL7 (chemokine ligand 7), CXCL1 (C-X-C motif chemokine 11) and IL-8 (interleukin 8). In turn, these cytokines increased tumour cell proliferation *in vitro*.

Fibroblasts have also shown to be a source of IGF-1 (insulin growth factor 1) in OSCC, which promotes tumour cell proliferation through activation of PI3K-AKT and Hedgehog signalling pathways [59]. The matrix-remodelling capabilities of CAF may also affect tumour proliferation [60]; CAF-secreted collagens, collagen8A1 and collagen11A1 [61], have been shown to modulate tumour cell growth through interaction with DDR1 (Discoidin domain receptor 1), which is overexpressed in HNSCC tissues.

## CAF, Angiogenesis, and the Metabolic Switch

Rapidly growing tumours create a hypoxic microenvironment and CAF play a major role in neo-angiogenesis, producing angiogenic factors such as VEGFA (vascular endothelial growth factor), and also attracting other cells, such as macrophages, which also contribute to the angiogenic process [62].

In OSCC, CAF are thought to be the main source of interleukin-6, which acts through an autocrine signalling loop to induce the secretion of VEGFA in CAF and also in OSCC cells [63]. Kayamori et al. [64] suggested that Notch signalling in CAF could also promote tumour angiogenesis; around one third of tongue OSCC cases were found to have CAF expressing NOTCH3 (neurogenic locus notch homolog protein 3) which correlated positively with tumour size and was associated with increased micro-vessel density.

CAF can also show metabolic adaptions to meet the energetic demands of tumour cells. In a phenomenon termed the “reverse Warburg effect,” CAF and tumour cells are metabolically coupled, whereby CAF metabolism is corrupted to undero aerobic glycolysis, producing metabolites such as lactate and pyruvate that can be used by cancer cells in oxidative phosphorylation. Lactate also has other pro-tumour effects, promoting angiogenesis, metastasis, and generating an immune-suppressive microenvironment [65–68]. Conversely, CAF have also been shown to promote glycolysis in tumour cells. Kumar et al. [3] reported that CAF-secreted HGF induces glycolysis in HNSCC cells and promotes expression of bFGF (basic fibroblast growth factor), which induces oxidative phosphorylation in fibroblasts creating another type of metabolic loop between the cells.

## CAF, Radiotherapy, and Chemotherapy

Radiotherapy with or without chemotherapy is commonly used in the treatment of OSCC. These treatments can modulate the tumour microenvironment in various ways. Notably, radiotherapy induces a wound healing response, promoting inflammation, CAF modulation/myofibroblast activation and ECM remodelling [69], and while CAF are considered relatively radioresistant [70], the DNA damage induced by radiation can result in a senescent phenotype, which can also be tumour promoting [71]. Kamochi et al. [72] showed that irradiated fibroblasts promote invasive growth of OSCC cells, upregulating expression of TGF-β1, that promote invasion and also potentiate further myofibroblasts differentiation. Indeed, irradiation is a potent activator of latent TGF-β and myofibroblasts which results in the fibrosis which is a well-recognised complication of radiotherapy [73]. CAF may also protect cancer cells from radiation. Huang et al. [74] found that radioresistant nasopharyngeal carcinoma had a higher infiltration of CAF compared with radiosensitive tissue, and showed that this radioprotective effect was modulated via IL-8/NF-κB signalling. This effect was diminished following treatment with Tranilast – a drug known to inhibit fibrosis and TGF-β signalling.

Chemotherapy, similar to radiotherapy, has been shown to promote the acquisition of CAF phenotype in breast [75], colorectal [76], and head and neck cancer [77]. A number of studies have also reported CAF-mediated chemotherapy resistance in HNSCC. CAF are associated with mediating resistance to cisplatin in head and neck cancer cells, and are generally more intrinsically resistant to this drug. Qin et al. [78] found that CAF transfer an exosomal miR-196a to cancer cells which renders them resistant to cisplatin through a downregulation of CDKN1B and ING5. Resistance to cisplatin has also been shown to be mediated by CAF-secreted collagens [61]; HNSCC cells pre-treated with collagen (or gelatin) prior to cisplatin treatment display significantly reduced cisplatin-induced apoptosis. In the presence of a DDR1 (Discoidin domain receptor 1; collagen receptor expressed by HNSCC cells) inhibitor, this protective effect was abolished.

Yegodayev et al. [79] also demonstrated that resistance to cetuximab is partially mediated by CAF. They found that TGF-β-activated CAF positively correlate with resistance to cetuximab both, *in vitr*o and *in vivo*, and inhibiting TGF-β signalling improved cetuximab treatment efficacy. In a separate study, CAF were shown to be non-sensitive to cetuximab, but their conditioned medium protected HNSCC cells from cetuximab in a dose-dependent manner [80]. This CAF-mediated resistance was suggested to be partially driven by an upregulation of metalloproteinases (MMPs) in both, CAF and HNSCC cells, following co-culture. MMP1 was the most upregulated protease, and use of its inhibitor partially restored the sensitivity of the HNSCC to cetuximab. Notably however CAF with silenced MMP-1 could still mediate cetuximab resistance, and the conclusion drawn from the study was that other MMPs (such as MMP-2,−3,−7,−13) were also likely involved in modulating this effect.

## CAF and Tumour Immune Suppression

The microenvironment of HNSCC is immunosuppressive and pro-inflammatory, and is associated with T-cell and NK-cell dysfunction, as well as accumulation of Tregs and pro-tumoral macrophages [81]. In recent years, the role of fibroblasts in promoting this suppressive microenvironment has generated much interest, particularly in the context of resistance to anti-PD-1/PD-L1 checkpoint immunotherapy. CAF contribute to tumour immune evasion through multiple mechanisms, affecting both innate and adaptive immunity [82]. Takahashi et al. [83] found that HNSCC CAF express elevated amounts of PD-L1 (also known as B7H1) and PD-L2 (B7DC); both molecules interact with PD-1 receptor on T-cells to suppress effector functions [84, 85]. CAF also suppress infiltration of CD8 T-cells into tumours; in part this may result from T-cell interactions with the extracellular matrix “barrier” secreted by myCAF [86]. The desmoplastic stroma produced by myCAF is rich in collagen, fibronectin and various proteoglycans (hyaluronan, versican), which have been shown to “trap” T-cells and inhibit T-cell motility [87]. A dense meshwork of collagen fibres has also been shown to limit T-cell penetration into tumours [88] and enhance matrix density; the protease-independent nature of T-cell amoeboid migration leads to contact guidance where T-cells follow a path-of-least-resistance along collagen fibres [89]. CD8 T-cells in myCAF-rich murine and human tumours have been shown to upregulate expression of CTLA-4, which may play a role in modulating T-cell interactions with ECM [23]. In a murine model of pancreatic cancer, CXCL12 (C-X-C motif chemokine ligand 12) produced by CAF has also been show to inhibit T-cell infiltration into tumour islets [90]. CAF-derived TGF-β and IL-6 also have recognised roles in suppressing T-cell responses [91]. Recently, a novel subpopulation of antigen presenting CAF that interact with CD4 T-cells have been identified in pancreatic carcinoma, suggesting even more complex CAF interactions in shaping the adaptive T-cell response [12].

CAF have also been reported to mediate T-cell suppression in OSCC indirectly through attraction and polarisation of macrophages [92]. Clinicopathological analysis has revealed a positive relationship between numbers of CAF and TAMs (tumour-associated macrophages) in OSCC tumour samples, and both cell types correlate with vascular invasion and TNM stage [92]. Several CAF-derived factors have been shown to recruit macrophages into tumours and polarise them towards an M2 tumour promoting phenotype, including CXCL12 and MCP-1 (monocyte chemotactic protein 1) [93, 94], with M2 macrophages having suppressive effect on T-cells mediated by increased expression of arginase I, interleukin-10 and TGF-β [92]. Similarly, CAF-secreted CCL7 that has been shown to increase invasion of OSCC cells is also chemotactic for macrophages [95].

The recruitment of immune cells by CAF is usually described in terms of soluble immunomodulatory factors, such as CXCL10 (C-X-C motif chemokine ligand 10), IL-6, MCP-1 [96], but recently a novel mechanism for recruitment of macrophages was proposed by Pakshir et al. [97], who demonstrated that macrophages can sense ECM deformation resulting from myofibroblast contraction, and migrate towards them. This mechanosensing mechanism could potentially attract macrophages into CAF-rich tumours independent of chemotactic factors.

## CAF-Targeting Strategies

**Figure 2:**
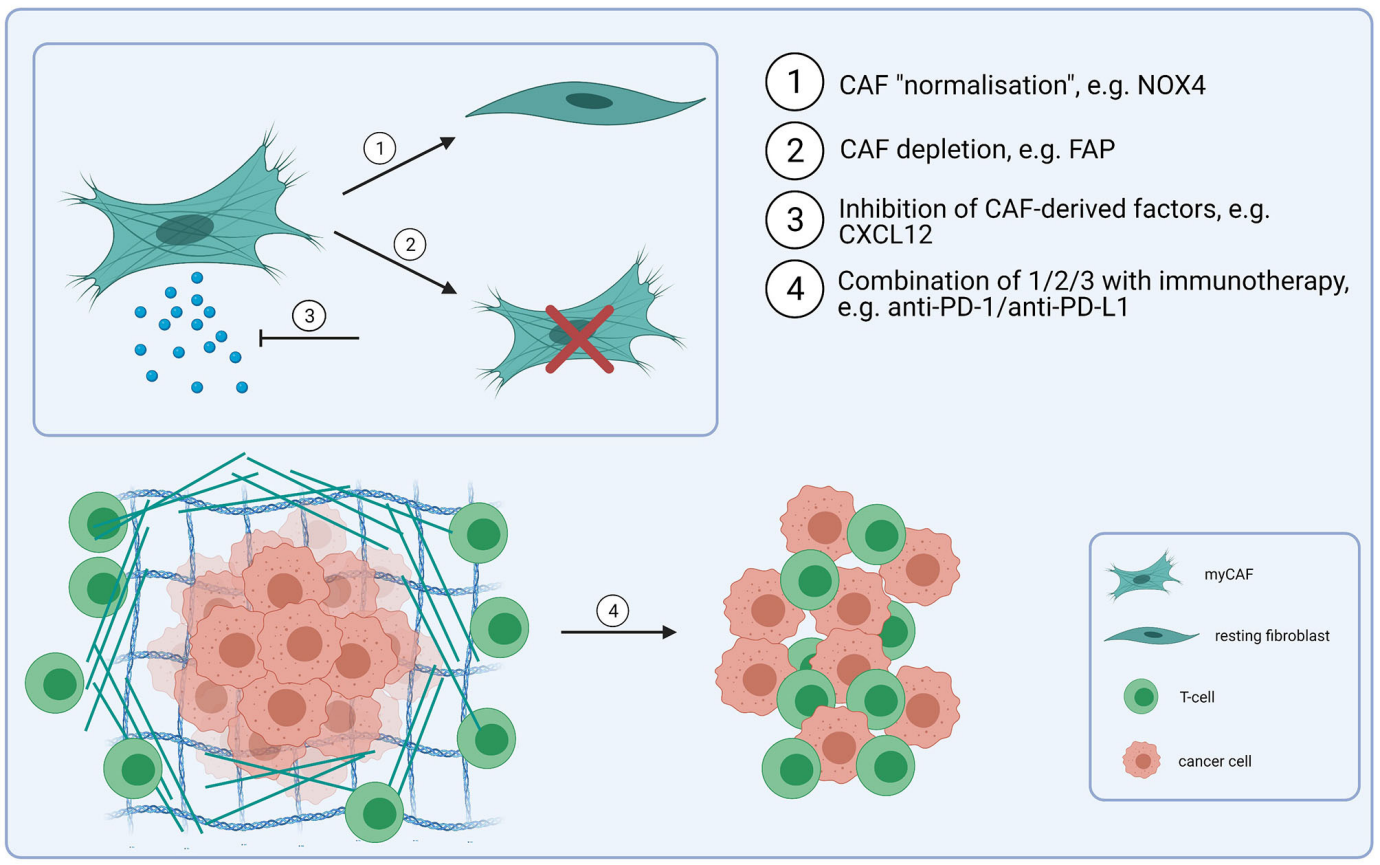
CAF-targeting strategies. Combining immunotherapy-based treatmens (e.g., anti-PD-1/anti-PD-L1) with CAF-targeting, either through CAF “normalisation” (1) (e.g., NOX4 inhibitors), depletion (2) (e.g., FAP-based depletion) or through disrupting a signalling network between CAF and cancer cells/T-cells (e.g., via inhibition of CXCL12) (3). These strategies could improve the response to checkpoint immunotherapy and result in an increased infilitration of T-cells into tumours (4) (Created with BioRender.com).

In addition to their tumour-promoting functions, CAF have been shown to confer resistance to different types of cancer therapy, including cetuximab and anti-PD-1/PD-L1 checkpoint immunotherapy, as well as radiotherapy and cisplatin chemotherapy [77–80, 98–101], suggesting that therapeutic CAF targeting could increase response rates for a diverse range of treatments. Potential strategies for CAF-directed therapy include inhibiting CAF activation or function, “normalizing” CAF or killing CAF within the tumour microenvironment (Figure 2), although clinical testing has generally yielded disappointing results [102, 103]. Given the myriad tumour promoting functions regulated by CAF, the specific context in which a particular pathway is targeted is clearly important. For example, FAP-expressing CAF have been shown to exclude T-cells from tumours through secretion of CXCL12 [90]; targeting the CXCL12/CXCR4 signalling axis using plerixafor (a CXCR4 inhibitor) has been shown to overcome this exclusion effect and promote response to anti-PD-1 [104]. Inhibiting CAF activation has also been investigated as a possible therapy; TGF-β signalling/mechanotransduction pathways are the primary signalling pathway regulating myCAF activation, although other growth factors and signalling molecules, including lysophosphatidic acid, PDGF, FGF, IL-6 and TNF (tumour necrosis factor) have also been implicated in the differentiation process. Targeting TGF-β is potentially problematic for several reasons; in the early stages of tumorigenesis it acts as a tumour suppressor; it also plays an important role in tissue homeostasis, and early testing of small molecule TGF-β1 inhibitors highlighted on-target cardiac toxicities and development of cutaneous carcinomas [105]. However, recognition of its role in promoting resistance to anti-PD-1/PD-L1 checkpoint immunotherapy through both CAF-dependent and independent mechanisms, has led to a resurgence in interest in targeting the pathway [106], including use of neutralising antibodies, receptor inhibitors and ligand traps [107]. With the identification of different fibroblast phenotypes and the recognition of CAF plasticity, “normalizing” CAF is an attractive concept, particularly since certain fibroblast phenotypes may be tumour suppressive [108]. In pancreatic cancer, vitamin D receptor has been shown to act as a master repressor of stellate cell activation, and treatment with calcipotriol (a synthetic derivative of calcitriol) reduces fibrosis and improves response to gemcitabine in tumour-bearing KPC mice [109]. Hanley et al. [22] identified NOX4 as a critical regulator of myCAF activation in multiple tumour types, including HNSCC, and found that inhibiting NOX4 using Setanaxib (GKT137831), a drug developed to treat organ fibrosis, suppressed myCAF activation and also “normalized” established myCAF. More recently, Ford et al. [23] developed myCAF-rich murine tumour models and showed that Setanaxib could be used to effectively overcome myCAF-mediated T-cell exclusion from tumours and potentiate response to anti-tumour vaccination and anti-PD-1 immunotherapies [23]. Grauel et al. [110] also examined the effect of TGF-β inhibition on the CAF phenotype *in vivo*, and found that TGF-β neutralisation reduced myCAF development and promoted an immunomodulatory phenotype characterised by strong response to interferon. CAF depletion could also be a therapeutic approach. Depletion of fibroblast activation protein (FAP)-positive CAF in murine models has been shown to enhance anti-tumour immunity [111]; Duperret et al. [112] used a DNA vaccine targeting FAP, which induced CD8+ and CD4+ T-cells and synergised with other tumour antigen-specific DNA vaccines to enhance anti-tumour immunity. However, FAP is widely expressed on different cell types [113] and there is still lack of a CAF-specific target that could make this approach effective.

## Conclusions

CAF support HNSCC progression and promote treatment resistance and have emerged as an attractive therapeutic target. Treatments designed to target CAF however, have not been successful clinically, and the identification of specific CAF targets has proven problematic, mostly due to a limited understanding of the molecular and functional phenotypes within a heterogenous CAF population and compounded by murine models that do not accurately recapitulate the stromal micorenvironment of human tumours. However, the advent of new technologies, particularly single-cell RNA-sequencing, is unpicking CAF complexity and there is great optimism in the field that effective CAF-targeted therapies are on the near horizon.

## Author Contributions

KB, CH, and GT wrote the manuscript. All authors contributed to the article and approved the submitted version.

## Conflict of Interest

The authors declare that the research was conducted in the absence of any commercial or financial relationships that could be construed as a potential conflict of interest.
